# Continuity of Care and Coordination of Care: Can they Be Differentiated?

**DOI:** 10.5334/ijic.6467

**Published:** 2023-02-17

**Authors:** Chi-Chen Chen, Yi-Chen Chiang, Yi-Chieh Lin, Shou-Hsia Cheng

**Affiliations:** 1 Department of Public Health, College of Medicine, Fu-Jen Catholic University, New Taipei City, Taiwan; 2 School of Public Health, Xiamen University, Xiamen, China; 3 Department of Public health, College of Health Sciences, Kaohsiung Medical University, Kaohsiung City, Taiwan; 4 Institute of Health Policy & Management, College of Public Health and Population Health Research Center, National Taiwan University, Taipei, Taiwan

**Keywords:** care continuity, care coordination, outpatient setting, questionnaire, psychometric analysis

## Abstract

**Introduction:**

Both care continuity and coordination are considered essential elements of health care system. However, little is known about the relationship between care continuity and coordination. This study aimed to differentiate the concepts of care continuity and coordination by developing and testing the reliability and validity of the Combined Outpatient Care Continuity and Coordination Assessment (COCCCA) questionnaire under the universal coverage health care system in Taiwan from a patient perspective.

**Methods:**

Face-to-face interviews were conducted nationwide with community-dwelling older adults selected via stratified multistage systematic sampling with probability-proportional-to-size process. A total of 2,144 subjects completed the questionnaire, with a response rate of 44.67%.

**Results:**

The 16 items of the COCCCA questionnaire were identified via item analysis and principal component analysis (PCA). The PCA generated five dimensions: three continuity-oriented (interpersonal, information sharing and longitudinal between patients and physicians) and two coordination-oriented (information exchange and communication/cooperation among multiple physicians). The second-order confirmatory factor analysis supported the factor structure and indicated that distinct constructs of care continuity and coordination can be identified.

**Conclusion:**

The COCCCA instrument can differentiate the concepts of care continuity and care coordination and has been demonstrated to be valid and reliable in outpatient care settings from a patient perspective.

## Introduction

Care continuity and care coordination are important features of a health care system. Many countries have introduced strategies to improve care continuity and coordination [1,2], for example, the patient-centered medical home, accountable care organization programs under the Affordable Care Act in the United States [1]. To investigate the impacts of these programs, good measurement tools for care continuity and care coordination are critical. Several studies have argued that the two concepts have distinct features [3,4,5,6,7]. However, the concepts of care coordination and care continuity are sometimes used interchangeably [8,9,10].

WHO (2018) provided the definition of care continuity as “the extent to which a series of discrete health care events is experienced by people as coherent and interconnected over time and consistent with their health needs and preferences.” [7] Before the year 2000, the concept of care continuity focused on the relationship between patients and their physicians [4,11,12,13,14,15] or uninterrupted health care [8]. Few studies adopted a multidimensional concept of continuity of care [16]. However, after 2000, the concept of care continuity was treated as multidimensional [17,18,19,20]. Three types of care continuity have been identified: longitudinal continuity [7,16,17,19], informational continuity [7,17,19] and interpersonal/relational continuity [16,17,18,19] between patients and their physicians. Longitudinal continuity is characterized by a regular source of care whereby patients receive most care from a provider or a team of providers for a long period of time [19]. This ongoing relationship existing between patients and health care providers allows physicians to be familiar with the medical history and current condition of patients. Informational continuity is defined as the transfer of a patient’s information across episodes of care, both between the patient and providers and among providers, through the organized collection of medical records as well as the memory of the physicians who establish a relationship with the patient (such as knowledge of the patient’s preferences) [19,20]. Interpersonal continuity represents patients’ trust that is established with their usual care providers from a personal perspective, as well as physicians’ view of themselves as having personal responsibility for patient care [19,20].

However, the growth of the elderly population with an increasing prevalence of chronic conditions and the increase in specialization among health care professionals have challenged the concept of care continuity. People are more likely to receive specialty care from various health care providers in different settings [21,22,23]. Researchers have suggested that when patients see multiple providers, the concept of care continuity should focus not only on the ongoing interpersonal continuity between patients and their physicians but also on care coordination among multiple physicians in different care settings, such as cross-boundary continuity [17] and managerial continuity [18]. The concepts of cross-boundary continuity and managerial continuity are similar to the concept of care coordination.

On the other hand, WHO (2018) provided the definition of care coordination as “a proactive approach to bringing together care professionals and providers to meet the needs of service users, to ensure that they receive integrated, person-focused care across various setting.” [7] In the early years, the concept of care coordination focused on primary care settings. It was defined as a provider’s recognition of information concerning a patient from one visit to another [24], other providers’ awareness of the involvement of the primary care physician [3] and the advanced coordination of patient care by the primary care physician [25]. Later, care coordination began to be considered a multidimensional concept and to frequently include information transfers among providers [26,27], communication among providers [7,26,27,28,29,30] and cooperation among providers to create a care plan for a patient [7,18,26,27]. Gradually, the concept of care coordination has been extended to the entire health care system instead of focusing only on primary care settings [31].

Measurements of care continuity and coordination were constructed by using medical records, claim data and patient- or provider-reported surveys. Freeman and colleagues (2001) proposed the evaluation of patients’ experiences of coordinated and smooth progression of care [17]; since then, measuring care continuity and coordination from a patient perspective has attracted attention [32]. After reviewing the literature, we categorized instruments measuring patients’ experiences of care continuity and care coordination into three categories. Instruments in the first category aim to evaluate the performance of primary care services from a patient perspective [28,33,34,35]. Some instruments in this category contain subscales to measure specific dimensions of care continuity and coordination, such as longitudinality [28,33,34,35], interpersonal communications [28] and coordination of care [28,33,34,35].

Instruments in the second category measure patient-reported care continuity. The majority of the instruments in this category aim to capture the multidimensional concept of care continuity proposed by Saultz (2003) [19] and Haggerty et al. (2003) [18], with different focuses [36,37,38,39,40,41]. For example, the Cuestionario Continuidad Asistencial Entre Niveles de Atencion (CCAENA) questionnaire aims to assess care continuity across different health care levels, including primary and secondary care providers [40]. Haggerty and colleagues (2012) developed a generic measure of multidimensional care continuity to be used when patients encounter several physicians [39]. In general, most of the care continuity instruments include both items concerning the “ongoing relationship between patients and specific physicians (e.g., longitudinal, interpersonal, and self-management information, etc.)” and items concerning “care coordination among different physicians (e.g., information gap, management, and cross-boundary continuity, etc.)” [36,37,38,39,40].

The third category of instruments aims to measure patient-reported care coordination, which is relatively new in this research field. McGuiness and Sibthiorpe (2003) developed the measurement tool specifically for care coordination [42]. The Agency for Healthcare Research and Quality (AHRQ) in the United States introduced a standardized definition of care coordination and later published the “Care Coordination Measures Atlas”. This atlas provides an overview of care coordination measurements as well as a framework of key dimensions that are important for measuring care coordination [43]. Afterward, based on the AHRQ atlas, Schultz et al. (2013) reviewed the existing care coordination measures of 96 instruments and found that the majority of the measures used survey data (88%) and focused on primary care settings (58%) [27].

In recent years, each care coordination measurement tool has had individual features, such as being patient centered [44], including patient and provider perspectives [45], and being applicable across different settings [46]. Similarly, some instruments for care coordination have included subscales of care continuity, such as information [44], interpersonal communication [42,44,45,46] and the longitudinal relationship between patients and their physicians [46].

In summary, we considered the concept of continuity of care to refer primarily to the interpersonal and longitudinal relationship and information sharing between patients and their physicians, which is similar to the definition of Saultz (2003) [19]. On the other hand, the concept of care coordination refers mainly to coordination among multiple health care providers, including information exchange and communication/cooperation. However, the existing instruments measuring care continuity include subscales of managerial continuity (similar to the concept of care coordination) [36,37,38,39], while some of the care coordination measures include subscales of care continuity, such as interpersonal/relational continuity [27,42,44,45,46]. It is academically valuable to distinguish between the constructs of care continuity and care coordination and identify the different dimensions of care continuity and care coordination described in the literature. To the best of our knowledge, no existing instrument measures both care continuity and care coordination as separate concepts and incorporates the corresponding dimensions under each concept. This study aims to differentiate the two concepts and to develop and validate a generic questionnaire of both care continuity and coordination from a patient perspective, namely, the Combined Outpatient Care Continuity and Coordination Assessment (COCCCA) questionnaire, by using a nationally representative random sample of community-dwelling older adults.

### Taiwan’s health care system

There is no formal referral requirement or primary care system in Taiwan’s health care system. Patients are free to visit specialists at community clinics or hospital outpatient departments based on their preferences [47]. In addition, Taiwan implemented a compulsory National Health Insurance (NHI) program in 1995 to provide health care coverage for all residents. Patients in Taiwan are often criticized for “doctor-hopping” behavior [48] and are likely to receive fragmented care from various health care providers. Therefore, we considered Taiwan’s universal health care system to be an ideal setting for measuring patient experiences of care continuity and care coordination.

## Research methods

In the development process, we referred to the development measures tutorial recommended by HinKin (2011) [49], which provides a straightforward guide for the development of scales.

### Item generation and development

We developed the COCCCA questionnaire in the following steps. First, we conducted a review of the relevant literature regarding the concepts of care continuity and coordination. We considered the concept of care continuity to consist of three dimensions: longitudinal continuity, information continuity and interpersonal continuity between patients and their physicians [18,19]. The considered the concept of care coordination to consist of two dimensions: information transfer and communication/cooperation among multiple physicians [18,26,27,28,29,30]. Second, after identifying the dimensions of both constructs, we reviewed the existing instruments to identify and select potential candidate items [28,34,35,36,37,38,39,40,42,44,45]. Third, we developed certain new items that we considered appropriate and necessary for inclusion in the COCCCA questionnaire. Finally, a total of 32 candidate items were generated and mapped to the corresponding dimensions in this study.

After the initial development of the draft COCCCA questionnaire, a pilot survey was conducted by face-to-face interviews with community-dwelling residents aged 50 years or older (N = 179 subjects). The pilot COCCCA questionnaire was found to be a reliable and valid instrument [50]. Based on the results of the pilot study, we held an expert meeting with eleven professionals in related fields. They provided comments on whether each item had the appropriate content to unambiguously assess the corresponding dimension and provided suggestions for rewording question items to improve their clarity. We also calculated an item-level content validity index by using a four-point scale (1 = very inappropriate; 2 = inappropriate; 3 = appropriate; 4 = very appropriate) for the experts’ assessment. Then, for each item, the content validity index at the item level was calculated as the number of experts giving a rating of either appropriate or very appropriate divided by the total number of experts [51]. In this study, the item-level CVI ranged from 0.7 to 1.0. According to the results of the pilot survey and the opinions of the experts, we then selected and modified the items for the questionnaire.

The final question items for the first version are shown in Supplementary Table 1. The questions concerning the experiences of care continuity and coordination were confined to the previous 12 months. The respondent experience referred to the health care services provided by their most frequently seen physicians in either community clinics or hospital outpatient departments. In this study, the majority of the respondents (76.54%) had a most frequently seen physician (or usual provider). If the subjects did not have the most frequently seen physician, they were asked about their overall experience in the previous year. The responses to each item were scored using a Likert scale with five response options.

**Table 1:** Characteristics of the total respondents and the respondents who visited at least two physicians.


CHARACTERISTICS	TOTAL RESPONDENTS		RESPONDENTS WHO VISITED AT LEAST TWO PHYSICIANS		P VALUE
	
	N = 2144		N = 1730		

Basic characteristics					

Sex (N, %)					0.817

Male	1064	49.63	865	50	

Female	1080	50.37	865	50	

Age (Mean, SD)	71.61	7.75	71.41	7.60	

Age groups (N, %)					0.673

60–69	1059	49.39	872	50.4	

70–79	711	33.16	574	33.18	

80+	374	17.44	284	16.42	

Individual income groups (N, %)					0.991

<NT10,000	944	44.03	760	43.93	

NT10,000–29,999	724	33.77	585	33.82	

NT30,000–49,999	167	7.79	136	7.86	

NT 50,000+	206	9.61	172	9.94	

Missing	103	4.8	77	4.45	

Level of education (N, %)					0.790

Illiterate/no formal education	243	11.33	188	10.87	

Primary school	846	39.46	665	38.44	

Junior high/senior high school	704	32.84	577	33.35	

College/university	350	16.32	299	17.28	

Missing	1	0.05	1	0.06	

Location of residence (N, %)					0.863

Rural area	434	20.24	354	20.46	

Urban	1708	79.66	1374	79.42	

Missing	2	0.09	2	0.12	

Health status					

Perceived health status (N, %)					0.218

Very good	204	9.51	135	7.8	

Good	783	36.52	608	35.14	

Fair	665	31.02	554	32.02	

Poor	403	18.8	352	20.35	

Very poor	86	4.01	78	4.51	

Missing	3	0.14	3	0.17	

Number of chronic conditions (Mean, SD)	1.41	1.33	1.51	1.35	

Number of chronic conditions (N, %)					0.077

0	646	30.13	471	27.23	

1	628	29.29	502	29.02	

2+	870	40.58	757	43.76	

Specific chronic condition (N, %)					

Hypertension	957	44.64	808	46.71	0.199

Hyperlipidemia	506	23.6	426	24.62	0.459

Diabetes mellitus	508	23.69	417	24.1	0.766

Heart disease	357	16.65	314	18.15	0.220

Cerebrovascular disease	86	4.01	74	4.28	0.679

Renal disease	140	6.53	131	7.57	0.206

Lung disease	109	5.08	94	5.43	0.627

Liver disease	84	3.92	75	4.34	0.515

Peptic ulcer	166	7.74	160	9.25	0.093

Malignancy	120	5.6	112	6.47	0.253

Others	393	18.33	358	20.69	0.240


### Sampling and questionnaire administration

The target population for the survey was community-dwelling residents aged 60 years or older with a household registration in Taiwan. We employed a stratified three-stage systematic sampling method in this study, similar to population-based national surveys in Taiwan [52,53]. In this study, all townships/districts of Taiwan were stratified into 19 strata according to demographic structure, ecological economics and geographic location. In the first stage, for each stratum, townships/districts were the primary sampling units and were chosen with probability proportional to their population sizes (PPS). In the second stage, for each selected township/district, lins and villages (the smallest administrative units in Taiwan) were the secondary sampling units and were also sampled with PPS. Finally, in the third stage, community-dwelling residents were the basic sampling units and were selected from each selected lin and village. A systematic sampling method was used in each of the sampling stages.

This study was approved by the Research Ethics Committee of National Taiwan University Hospital in Taiwan (No. 201603076RINA). The randomly selected subjects were invited to participate in the survey via exclusively face-to-face interviews conducted at their homes by trained interviewers. Informed consent was obtained from each participant prior to the questionnaire interview. Because this study aimed to understand the experience of care continuity and care coordination from a patient perspective, patients who had no physician visits in the 12 months prior to the survey were excluded from the sample.

### Statistical analyses

To examine the psychometric properties of the COCCCA questionnaire, we conducted a variety of analyses using SAS statistics software package version 9.3 and Mplus version 7. We conducted item reduction and refined the questionnaire by using item analysis and principal component analysis (PCA) (Supplementary Table 1 and Supplementary Table 2). After the item reduction process, a final model was built for the items and their corresponding dimensions. A PCA with varimax rotation was again employed. The latent constructs of care continuity and coordination were simultaneously included in the confirmatory factor analysis (CFA) for the final model. Furthermore, we employed a second-order CFA that consisted of three levels: the secondary factor (i.e., the two latent constructs of care continuity and coordination) and the primary factor (i.e., the dimensions of interpersonal continuity, information sharing continuity and longitudinal continuity under the continuity construct and the dimensions of information exchange and communication/cooperation under the coordination construct). This CFA analysis tested the model as hypothesized for care continuity and coordination and demonstrated the distinctiveness of the constructs measured in this study. Several goodness-of-fit indexes of the second-order CFA were evaluated. For the comparative fit index (CFI) and Tucker-Lewis index (TLI), values >0.90 represented a good model fit. For the root mean square error of approximation (RMSEA), a value <0.05 indicated an excellent model fit, and a value <0.08 indicated an acceptable model fit. For standardized root mean square residual (SRMR), a value <0.05 indicated a good model fit [54].

In addition, the criterion-related validity of the care continuity and coordination constructs was examined based on the relationship between the constructs and their criterion measure. For care continuity, we assessed whether the patients had a usual provider of care as the criterion measure [55]. For care coordination, we employed a single item from the overall assessment of care coordination, i.e., “whether the patients perceive lack of care coordination”, as the criterion measure. Independent Student’s t-test (equal variances not assumed) was employed to determine whether significant associations existed between the items for each dimension and their criteria. Finally, the internal consistency of the three dimensions of care continuity and two dimensions of care coordination that emerged from the final model was then assessed by Cronbach’s alpha. A Cronbach’s alpha value ranging from 0.70 to 0.95 was considered acceptable [56].

### Sensitivity analyses

In this study, we conducted two sensitivity analyses to improve the robustness of our results. First, in addition to PCA with orthogonal varimax rotation, we employed PCA with direct oblimin or promax oblique rotation to allow for correlation among the dimensions. Second, we drew a sample of 500 subjects by using a simple random sampling scheme (i.e., the first group), and the remaining sample (1,023 respondents) served as the second group. Data from the first group were used for model building by conducting item reduction and PCA. Then, the second group was used for model confirmation through a second-order CFA model.

## Results

### Demographic characteristics and health status of the participants

A total of 2,144 subjects completed the questionnaire, for a response rate of 44.67% from November 19, 2018, to January 25, 2019. Respondents who had seen at least two physicians in the previous 12 months were included in the analysis (N = 1,730) because this study aimed to explore patients’ experience of care coordination among multiple physicians. The basic characteristics of the target population, the total number of respondents to the survey (N = 2,144) and the number of respondents who had visited at least two physicians (n = 1,730) are shown in Supplementary Table 3. Table 1 shows the demographic characteristics and health status of the total respondents (n = 2,144) and the respondents who had visited at least two physicians (n = 1,730). Among the total respondents, 49.63% were male, the mean age was approximately 72 years old. Half of respondents (49.16%) had graduated from junior high school or higher, while approximately 11% of respondents had no formal education. With regard to health status, 22.81% of respondents reported that their health status was very poor or poor, and 40.58% of the respondents reported having two or more chronic conditions (Table 1). Furthermore, we found no significant differences in demographic characteristics or health status measures between the total sample of respondents and those who had visited at least two physicians. However, slightly higher proportions of the respondents who had visited at least two physicians had a poor/very poor health status (24.86% vs. 22.81%) and had two or more chronic conditions (43.76% vs. 40.58%).

### Item reduction: item analysis and PCA

Supplementary Table 1 shows the excluded items and the criteria applied for item reduction. After the item reduction process, we performed PCA and found that the sample data were adequate for PCA (with a KMO value = 0.82). The PCA generated five factors for the remaining items that explained 59.30% of the variance. However, we found that the two items “Does the doctor usually listen to you with patience?” and “Does this doctor respect your opinion during the diagnosis and treatment process?” both loaded onto the information continuity dimension and the interpersonal continuity dimension. Because the factor loadings of these two dimensions were nearly the same (0.46 and 0.45, respectively, for the former item; 0.41 and 0.40, respectively, for the latter item), the two items were both removed from the model (table not shown). In summary, there were 26 items in the original COCCCA questionnaire, and 10 items were deleted in the process of item analysis and PCA. Therefore, the final COCCCA instrument consisted of 16 items, with 10 items for the care continuity construct (2 items for longitudinal continuity; 4 items for information sharing continuity between patient and physician; 4 items for interpersonal continuity) and 6 items for the care coordination construct (3 items for information exchange between patient and physician and 3 items for communication and cooperation among multiple physicians).

### Validity and reliability evaluation

Among the study subjects (n = 1,730), only those who responded to all 16 items in the COCCCA questionnaire were included in the validity and reliability analysis (n = 1523; 88.03%). Table 2 shows the mean and standard deviation of each item score, the item-total correlation coefficient, and the factor loading according to the five dimensions in the final version of the COCCCA questionnaire after the item reduction process. For the care continuity construct, the mean scores of the items in the dimensions of longitudinal, information transfer and interpersonal continuity ranged from 3.66 to 4.54. For the care coordination construct, the mean scores of the items in the dimensions of information exchange and communication/cooperation among multiple physicians ranged from 1.20 to 2.68.

**Table 2:** The COCCCA core items: Descriptive results, internal consistency and PCA results (N = 1523).


DIMENSION/CORE ITEMS	DESCRIPTIVE ANALYSIS		INTERNAL CONSISTENCY		FACTOR LOADING FROM PCA ANALYSIS				
		
	MEAN	SD	ITEM-TOTAL CORRELATION	CRONBACH’S ALPHA	FACTOR 1	FACTOR 2	FACTOR 3	FACTOR 4	FACTOR 5

Care continuity									

Longitudinal relationship between patient and doctor				0.92					

How long have you been receiving health care at the most frequently visited place?	4.39	1.19	0.86		0.07	–0.03	0.05	0.04	0.88

How long have you been seeing the most frequently visited doctor?	3.76	1.62	0.93		0.18	–0.03	0.09	0.00	0.84

Information sharing between patient and doctor				0.83					

Does this doctor clearly understand your health needs?	4.54	0.85	0.58		0.18	–0.01	0.56	–0.07	0.35

Does this doctor clearly explain your conditions or diagnosis to you?	4.38	1.06	0.70		0.26	0.04	0.66	0.02	0.23

Does this doctor clearly explain issues regarding medication usage to you?	3.72	1.56	0.79		0.13	0.07	0.75	0.14	–0.07

Does this doctor teach you how to take care of your medical conditions or treatment problems after you go home?	3.66	1.51	0.77		0.13	0.03	0.74	0.12	–0.06

Interpersonal relationship between patient and doctor				0.89					

Does this doctor show a friendly attitude during the visits?	4.45	0.67	0.73		0.65	0.05	0.26	0.03	0.11

Do you trust the professional competence of this doctor?	4.35	0.64	0.81		0.81	0.04	0.12	0.08	0.12

Does this doctor care about you?	4.15	0.74	0.82		0.79	0.09	0.13	0.14	0.04

Does this doctor make the best decision for your health care?	4.18	0.68	0.79		0.78	0.07	0.16	0.11	0.08

Care coordination									

Information transfer among doctors				0.85					

Do you actively tell this doctor about other physician visits you have elsewhere?	2.66	1.71	0.80		0.05	0.04	0.04	0.78	–0.01

Does this doctor actively ask you about other physician visits you have elsewhere?	1.83	1.35	0.76		0.11	0.18	0.10	0.75	–0.04

Does this doctor know the situation of other physician visits you have elsewhere?	2.68	1.45	0.75		0.13	0.14	0.06	0.72	0.08

Communication/cooperation among doctors				0.89					

Does this doctor communicate with other doctors about your care?	1.36	0.97	0.87		0.06	0.83	0.10	0.15	–0.04

Does this doctor tell you that he/she has discussed your medical conditions with other doctors?	1.25	0.83	0.86		0.12	0.85	0.02	0.11	–0.05

Does this doctor work together with other doctors about your care?	1.20	0.75	0.75		0.04	0.76	–0.01	0.10	0.02


COCCCA, Combined Outpatient Care Continuity and Coordination Assessment; PCA, principal component analysis; SD, standard deviation.

### PCA and CFA results

**Figure 1:**
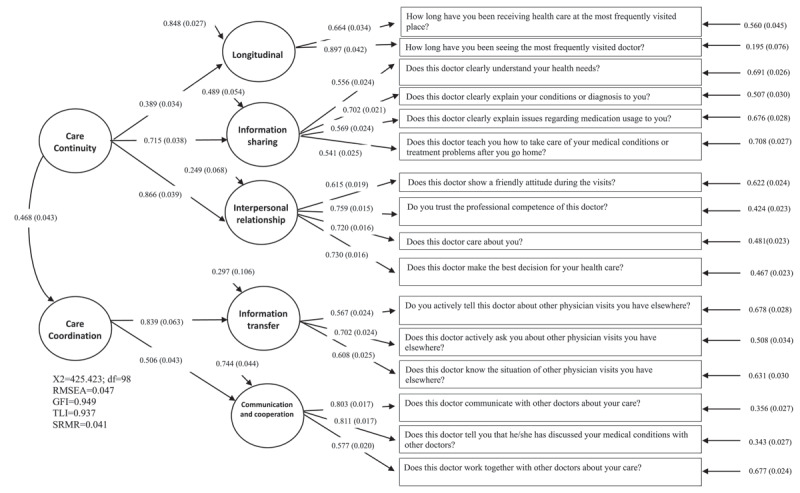
Confirmatory factor analysis of care continuity and care coordination.

We performed exploratory factor analysis by PCA on the final version of the COCCCA questionnaire with 16 items, and the sample data were adequate for PCA (KMO value = 0.80). The PCA generated five factors that explained 63.27% of the variance. We found that all of the factor loadings were larger than 0.4. The continuity-oriented dimensions included “interpersonal continuity” (factor 1), “information-sharing continuity” (factor 3), and “longitudinal continuity” (factor 5) between patients and their physicians. In addition, the coordination-oriented dimensions included “information exchange among multiple physicians” (factor 4) and “communication or cooperation among multiple physicians” (factor 2). The structure of the five factors was generally consistent with the a priori assumption and the results of the pilot study, and all of the items loaded onto the factors originally proposed for them (Table 2). Figure 1 shows the second-order CFA model, which confirms the factor structure from the final version of the COCCCA questionnaire. The CFA model in the analysis shows an excellent fit to the data. The goodness-of-fit statistics were *χ*^2^ = 425.423 (df = 98), RMSEA = 0.047, GFI = 0.949, TLI = 0.937 and SRMR = 0.041. In addition, the modest correlation between the two constructs of care continuity and care coordination (r = 0.468) indicated that care continuity and coordination are unique and distinct constructs.

### Criterion validity

Table 3 shows the association between the COCCCA items and the corresponding criterion-related measures. We found that the subjects who had a usual provider had significantly higher scores on all items of the three continuity dimensions. Similarly, we observed that the subjects who had experienced a lack of coordination tended to have lower scores on all of the items of the two coordination dimensions. Thus, the predictive validity of the care continuity and coordination construct was confirmed.

**Table 3:** Relationship between COCCCA items and the corresponding criterion-related measures.


DIMENSION/CORE ITEMS	HAVING A USUAL DOCTOR					PERCEIVED LACK OF CARE COORDINATION				
	
	NO (N = 312)		YES (N = 1211)		P VALUE	NO (N = 823)		YES (N = 527)		P VALUE
			
	MEAN	SD	MEAN	SD		MEAN	SD	MEAN	SD	

Care continuity										

Total score for longitudinal relationship between patient and doctor	4.25	1.83	9.16	1.49	<0.001	–	–	–	–	–

How long have you been receiving health care at the most frequently visited place?	3.25	1.83	4.68	0.71	<0.001	–	–	–	–	–

How long have you been seeing the most frequently visited doctor?	1.00	0.00	4.48	0.92	<0.001	–	–	–	–	–

Total score for information sharing between patient and doctor	14.64	4.41	16.73	3.24	<0.001	–	–	–	–	–

Does this doctor clearly understand your health needs?	4.07	1.21	4.66	0.68	<0.001	–	–	–	–	–

Does this doctor clearly explain your conditions or diagnosis to you?	3.82	1.37	4.52	0.91	<0.001	–	–	–	–	–

Does this doctor clearly explain issues regarding medication usage to you?	3.35	1.64	3.82	1.52	<0.001	–	–	–	–	–

Does this doctor teach you how to take care of your medical conditions or treatment problems after you go home?	3.40	1.53	3.73	1.49	0.001	–	–	–	–	–

Total score for interpersonal relationship between patient and doctor	15.88	2.17	17.45	2.02	<0.001	–	–	–	–	–

Does this doctor show a friendly attitude during the visits?	4.13	0.75	4.53	0.62	<0.001	–	–	–	–	–

Do you trust the professional competence of this doctor?	4.01	0.66	4.44	0.60	<0.001	–	–	–	–	–

Does this doctor care about you?	3.86	0.77	4.22	0.71	<0.001	–	–	–	–	–

Does this doctor make the best decision for your health care?	3.88	0.70	4.26	0.65	<0.001	–	–	–	–	–

Care coordination										

Total score for information transfer among doctors	–	–	–	–	–	7.56	3.60	6.82	3.19	<0.001

Do you actively tell this doctor about other physician visits you have elsewhere?	–	–	–	–	–	2.77	1.70	2.58	1.70	0.048

Does this doctor actively ask you about other physician visits you have elsewhere?	–	–	–	–	–	1.94	1.42	1.70	1.24	0.001

Does this doctor know the situation of other physician visits you have elsewhere?	–	–	–	–	–	2.84	1.47	2.54	1.39	<0.001

Total score for communication/cooperation among doctors	–	–	–	–	–	3.97	2.33	3.63	1.84	0.003

Does this doctor communicate with other doctors about your care?	–	–	–	–	–	1.42	1.07	1.28	0.82	0.004

Does this doctor tell you that he/she has discussed your medical conditions with other doctors?	–	–	–	–	–	1.30	0.90	1.19	0.72	0.014

Does this doctor work together with other doctors about your care?	–	–	–	–	–	1.25	0.83	1.16	0.63	0.031


COCCCA, Combined Outpatient Care Continuity and Coordination Assessment; PCA, principal component analysis.

### Reliability analyses

The Cronbach’s alpha coefficients ranged from 0.83 to 0.92 for the three dimensions of the care continuity construct and were 0.85 and 0.89 for the two dimensions of the care coordination construct. We found that the five dimensions of the COCCCA had acceptable internal consistency (Table 2).

### Results of sensitivity analyses

In the first sensitivity analysis, we found that there were no differences in the factor structures between the orthogonal varimax and direct oblimin or promax oblique rotated solutions (Supplementary Table 2). Second, we randomly selected 500 subjects (of 1523 respondents) for model building, including item analysis and PCA, and the remaining 1023 subjects were used for the CFA model. The results of these sensitivity analyses were similar to those of the original analyses (Supplementary Table 4 and Supplementary Figure 1).

## Discussion

### Summary

This study aimed to differentiate the concepts of care continuity and care coordination by developing and testing the reliability and validity of the COCCCA questionnaire among subjects aged 60 years or older. This measurement tool appears to be a concise, reliable and valid instrument that can be applied to measure the multidimensional concept of both care continuity and care coordination from a patient perspective. In addition, the results indicated that distinct dimensions of care continuity and care coordination can be identified.

### Strengths and limitations

The limitations of this study should be mentioned. First, the response rate to our survey was relatively low (44.67%); however, it was similar to those in other surveys on patient experience of care continuity or care coordination, which ranged from 22.5% to 48% [36,39,40,41,45], except the response rates reported by Uijen and colleagues in 2011 and 2012 of 72% and 76%, respectively [37,38]. In addition, there were slight differences in age and sex between the target population aged 60 years and above and the study sample, so generalizations should be made with caution. Second, patient experience measures of care continuity and care coordination might have been affected by patient preferences or health literacy, which were not considered in this study. Third, care coordination activities between physicians usually could not be observed by the patients, as has been noted by previous researchers [39]. Finally, the COCCCA instrument focused on care coordination among different physicians (or specialists) in the outpatient setting (or ambulatory care setting) only. Therefore, the COCCCA instrument might not be applicable across different settings, such as in hospitals or long-term care.

A strength of this study is that we used a nationwide representative random sample of older adults sampled via stratified multistage systematic sampling with PPS to develop the instrument. A number of instruments have been developed based mainly on specific groups of subjects recruited for the research, such as respondents invited by a limited number of general practitioners [36,37,38,39,40], subjects recruited in hospital outpatient departments [38] and subjects recruited in community health centers in certain geographic areas [45]. In other studies, patients have been recruited by random sampling in certain practice settings [36,40,45]. Therefore, the results from this study may be more representative than those from previous studies that were based on subjects who were recruited by health care providers or were recruited in limited geographic areas. In addition, existing instruments measuring care continuity or care coordination tended to include the other as part of the concept, thus treated as the subscales, and each instrument has individual features. The COCCCA instrument developed in this study measures both care continuity and care coordination as separate concepts and incorporates the corresponding dimensions under each concept.

### Comparison with previous studies

In general, the findings of this study support the argument of previous researchers that both care continuity and care coordination are multidimensional concepts [16,18,19]. The COCCCA instrument consists of five dimensions from the patient’s perspective: three dimensions (longitudinal continuity, information sharing continuity and interpersonal relationship continuity between patients and physicians) under the care continuity construct and two dimensions (information exchange and communication/coordination among multiple physicians) under the care coordination construct.

Regarding the longitudinal dimension of care continuity, Saultz considered longitudinal continuity to be a dimension of care continuity [19]. However, Haggerty considered the longitudinal aspect to be not a dimension of care continuity but an intrinsic part of continuity [18]. In this study, we found that longitudinal continuity is one of the dimensions under the care continuity construct, which is consistent with other reports [41,57]. In addition, the majority of the existing instruments have included the aspects of interpersonal or relational continuity [28,36,37,38,39,40]. Previous researchers have argued that interpersonal continuity is the most important aspect of care continuity [19,20]. In this study, we found that the interpersonal dimension is highly correlated with the continuity construct compared with the longitudinal and information transfer dimensions.

Regarding the dimension of information transfer or exchange, in this study, we demonstrated that there are two types of information transfer [58], including information sharing between patients and their physicians under the care continuity construct and information exchange among multiple physicians under the care coordination construct. Information sharing between patients and their physicians has been identified in previous studies as relational continuity [40] or information continuity [39,40,59]. Notably, we considered the importance of patient engagement in care continuity [39] by incorporating the item “Does the doctor teach you how to take care of your medical conditions or treatment problems after you go home?” in the information dimension, which indicates that knowledge transferred from the physician might empower the patient’s self-care ability at home.

Information exchange among multiple physicians was identified as one dimension of care coordination in this study. Previous researchers have considered information transfer among physicians to be important in achieving care coordination [18,36] and several studies have included the dimension of information exchange between physicians [39,40]. In a health system without primary care physicians, such as that in Taiwan, we suggest that no physician is responsible for information transfer among physicians. Therefore, we developed a new item, “Do you actively tell the doctor about other physician visits you have elsewhere?,” which implies the importance of patient engagement in care coordination among multiple physicians. The inclusion of this item was supported by a previous study that revealed that patient engagement is associated with a lower likelihood of care coordination problems [60].

Finally, the dimension of communication/cooperation among physicians in this study is similar to the dimensions of managerial continuity [36,39,40], team and cross-boundary continuity [36,37,38] and care coordination [28,35]. The items in this dimension measure adequate communication or cooperation between physicians on behalf of the patient. Interestingly, compared to the dimensions of care continuity, the dimension of communication/cooperation among physicians has a negatively skewed distribution (with a mean value lower than 2). This may be because patients cannot easily observe physicians’ efforts to communicate or cooperate with other physicians. We believe that it would be valuable to measure the degree of care coordination on the basis of positive and negative items as well as from the perspectives of both patients and their physicians in future studies [45].

## Conclusion

This study developed the COCCCA instrument for measuring care continuity and care coordination by using a nationwide representative sample of community-dwelling older adults. The COCCCA instrument differentiates the concepts of care continuity and care coordination, and it was demonstrated to be valid and reliable. The instrument reliably assesses care continuity and coordination simultaneously in outpatient care settings from a patient perspective. Future studies are recommended to examine the agreement of patient-reported and claim-based care continuity and care coordination measures by involving general population and employing advanced scoring instruments, and to investigate the effects of both types of measures on patient health outcomes.

## Additional File

The additional file for this article can be found as follows:

10.5334/ijic.6467.s1 Supplemental file. Supplementary Tables 1 to 4 and Supplementary Figure 1.
