# Supplementary material for: Continuity of Care and Coordination of Care: Can they Be Differentiated?
Source: Int J Integr Care. 2023 Feb 17;23(1):10. doi: 10.5334/ijic.6467 (PMC9936907; doi:10.5334/ijic.6467)
Supplement: Supplemental file. — Supplementary Tables 1 to 4 and Supplementary Figure 1. [file ijic-23-1-6467-s1.pdf]

Supplementary Table 1 Results of the item reduction process with the criteria for exclusion

Constructs/dimensions/items	Criteria					Excluded item	Reasons for exclusion
	Criterion 1:	Criterion 2:	Criterion 3:	Criterion 4:	Criterion 5: Sizable		
**Care continuity**							
**Longitudinal continuity between patients and physician**							
How long have you been receiving health care at the most	-	-	-	-	-		
How long have you been seeing the most frequently visited	-	-	-	-	-		
**Information transfer between patient and physician**							
Is this doctor aware of your medical history?	-	7.46%	-	-	-	X	Relatively high missing rate
Does this doctor clearly understand your health needs?	-	-	-	-	-		
Does this doctor clearly explain your conditions or diagnosis to you?	-	-	-	-	-		
Does this doctor clearly explain issues regarding medication usage	-	-	-	-	-		
Does this doctor teach you how to take care of your medical	-	-	-	-	-		
**Interpersonal relationship between patient and physician**							
Does this doctor show a friendly attitude during the visits?	-	-	-	-	-		
How much time does this doctor usually spend on you for a visit?	-	-	r=0.48	-	-	X	Low item-total correlation/Cronbach's alpha
Does this doctor usually listen to you with patience?	-	-	-	-	-	X	Item loaded on multiple factors for PCA
Does this doctor always answer your questions concerning your health	-	6.94%	-	-	-	X	Relatively high missing rate
Do you always understand the doctor’s answers?	-	-	r=0.46	-	-	X	Low item-total correlation/Cronbach's alpha
Does this doctor pay attention to your feelings during the diagnosis	-	5.32%	-	-	-	X	Relatively high missing rate
Does this doctor respect your opinion during the diagnosis and	-	-	-	-	-	X	Item loaded on multiple factors for PCA
Do you trust the professional competence of this doctor?	-	-	-	-	-		
Does this doctor care about you?	-	-	-	-	-		
Does this doctor make the best decision for your health care?	-	-	-	-	-		
**Care coordination**							
**Information exchange among multiple physicians**	-	-	-	-	-		
Do you actively tell this doctor about other physician visits you have	-	-	-	-	-		
Does this doctor actively ask you about other physician visits you have	-	-	-	-	-		
Does this doctor know the situation of other physician visits you have	-	-	-	-	-		
Has this doctor ever reviewed your NHI Medi-Cloud information and	-	-	-	-	0.61→0.69	X	Cronbach's alpha increase
**Communication and cooperation among multiple physicians**							
Does this doctor communicate with other doctors about your care?	-	-	-	-	-		
Does this doctor tell you that he/she has discussed your medical	-	-	-	-	-		
Has you received conflicting medical advice from different doctors in	-	-	r=0.31	-	0.53→0.57	X	Low item-total correlation/Cronbach's alpha
Has you received repeated tests or examinations from different doctors	-	-	-	-	0.53→0.57	X	Cronbach's alpha increase
Does this doctor work together with other doctors about your care?	-	-	-	-	-		

Supplementary Table 2 The factor loadings of PCA analysis with oblique rotation

	Direct Oblimin					Promax				
	Factor1	Factor2	Factor3	Factor4	Factor5	Factor1	Factor2	Factor3	Factor4	Factor5
**Care continuity**										
**Longitudinal continuity between patients and doctor**										
How long have you been receiving health care at the most frequently visited place?	0.19	-0.03	0.13	0.03	0.88	0.19	-0.03	0.13	0.03	0.88
How long have you been seeing the most frequently visited doctor?	0.29	-0.03	0.18	0.01	0.86	0.29	-0.03	0.19	0.01	0.86
**Information sharing between patient and doctor**										
Does this doctor clearly understand your health needs?	0.32	0.02	0.60	-0.03	0.43	0.32	0.02	0.60	-0.02	0.43
Does this doctor clearly explain your conditions or diagnosis to you?	0.41	0.09	0.71	0.09	0.33	0.41	0.09	0.72	0.09	0.33
Does this doctor clearly explain issues regarding medication usage to you?	0.29	0.14	0.77	0.21	0.02	0.28	0.14	0.77	0.22	0.02
Does this doctor teach you how to take care of your medical conditions or treatment problems after you go home?	0.28	0.09	0.75	0.18	0.02	0.27	0.09	0.75	0.19	0.02
**Interpersonal relationship between patient and doctor**										
Does this doctor show a friendly attitude during the visits?	0.70	0.12	0.39	0.12	0.22	0.70	0.13	0.41	0.13	0.23
Do you trust the professional competence of this doctor?	0.83	0.13	0.29	0.17	0.24	0.83	0.13	0.31	0.18	0.25
Does this doctor care about you?	0.81	0.18	0.29	0.23	0.16	0.81	0.18	0.31	0.24	0.17
Does this doctor make the best decision for your health care?	0.80	0.15	0.32	0.20	0.119	0.80	0.15	0.33	0.21	0.20
**Care coordination**										
**Information transfer among doctors**										
Do you actively tell this doctor about other physician visits you have elsewhere?	0.14	0.15	0.11	0.78	-0.02	0.14	0.16	0.12	0.78	-0.01
Does this doctor actively ask you about other physician visits you have elsewhere?	0.22	0.30	0.18	0.78	-0.04	0.22	0.30	0.19	0.78	-0.04
Does this doctor know the situation of other physician visits you have elsewhere?	0.23	0.25	0.14	0.74	0.08	0.23	0.25	0.15	0.74	0.09
**Communication/cooperation among doctors**										
Does this doctor communicate with other doctors about your care?	0.15	0.85	0.15	0.27	-0.05	0.15	0.85	0.17	0.28	-0.06
Does this doctor tell you that he/she has discussed your medical conditions with other doctors?	0.19	0.86	0.08	0.24	-0.06	0.19	0.86	0.10	0.25	-0.06
Does this doctor work together with other doctors about your care?	0.11	0.76	0.04	0.21	0.00	0.11	0.76	0.05	0.21	-0.01

Supplementary Table 3 Basic characteristics of the target population, total respondents and respondents who visited at least two physicians

Characteristics	Target population (age>=60		Total respondents to the survey			Respondents who visited at least two		
	N=4,849,818		N=2,144		P value	N=1,730		P value
	N	%	N	%		N	%	
Sex (N, %)					<0.05			<0.05
Male	2,266,624	46.7	1,064	49.63		865	50.00	
Female	2,583,194	53.3	1,080	50.37		865	50.00	
Age groups (N, %)					<0.05			<0.05
60-69	2,803,961	57.8	1,059	49.39		872	50.40	
70-79	1,278,868	26.4	711	33.16		574	33.18	
80+	766,989	15.8	374	17.44		284	16.42	
Level of education (N, %)					>0.05			>0.05
Illiterate/no formal education/primary school	2,396,119	49.4	1,089	50.79		853	49.31	
Junior high school/senior high school	1,643,451	33.9	704	32.84		577	33.35	
College/university	810,248	16.7	350	16.32		299	17.28	

Supplementary Table 4 COCCCA items: Descriptive analysis, internal consistency and PCA results for the first group of 500 respondents

Constructs/dimensions/items	Descriptive analysis			Internal consistency		Factor loading from PCA analysis*				
	N	Mean	SD	Item-total	Cronbach's	Factor 1	Factor 2	Factor 3	Factor 4	Factor 5
**Care continuity**										
**Longitudinal continuity between patients and doctor**					0.74					
How long have you been receiving health care at the most frequently visited place?	500	4.40	1.18	0.85		0.05	-0.01	0.04	0.87	0.05
How long have you been seeing the most frequently visited doctor?	500	3.80	1.60	0.92		0.17	-0.03	0.04	0.82	0.03
**Information sharing between patient and doctor**					0.72					
Does this doctor clearly understand your health needs?	500	4.51	0.90	0.60		0.26	0.00	0.45	0.49	-0.09
Does this doctor clearly explain your conditions or diagnosis to you?	500	4.36	1.10	0.72		0.24	0.05	0.62	0.36	0.04
Does this doctor clearly explain issues regarding medication usage to you?	500	3.70	1.57	0.80		0.11	0.09	0.80	0.01	0.16
Does this doctor teach you how to take care of your medical conditions or	500	3.73	1.47	0.80		0.16	0.05	0.81	-0.03	0.10
**Interpersonal relationship between patient and doctor**					0.81					
Does this doctor show a friendly attitude during the visits?	500	4.40	0.70	0.72		0.66	0.06	0.20	0.06	0.04
Do you trust the professional competence of this doctor?	500	4.31	0.66	0.83		0.82	0.04	0.08	0.16	0.10
Does this doctor care about you?	500	4.12	0.75	0.84		0.79	0.13	0.18	0.07	0.14
Does this doctor make the best decision for your health care?	500	4.15	0.71	0.81		0.79	0.07	0.10	0.12	0.14
**Care coordination**										
**Information transfer among doctors**					0.66					
Do you actively tell this doctor about other physician visits you have elsewhere?	500	2.65	1.74	0.79		0.14	0.01	0.06	-0.06	0.75
Does this doctor actively ask you about other physician visits you have elsewhere?	500	1.86	1.43	0.77		0.07	0.22	0.11	0.03	0.75
Does this doctor know the situation of other physician visits you have elsewhere?	500	2.69	1.45	0.75		0.12	0.15	0.06	0.09	0.73
**Communication/cooperation among doctors**					0.78					
Does this doctor communicate with other doctors about your care?	500	1.37	0.97	0.89		0.08	0.87	0.11	-0.07	0.14
Does this doctor tell you that he/she has discussed your medical conditions with	500	1.24	0.83	0.86		0.12	0.86	0.03	-0.05	0.10
Does this doctor work together with other doctors about your care?	500	1.22	0.81	0.75		0.06	0.72	0.03	0.07	0.12

COCCCA, Combined Outpatient Care Continuity and Coordination Assessment; PCA, principal component analysis

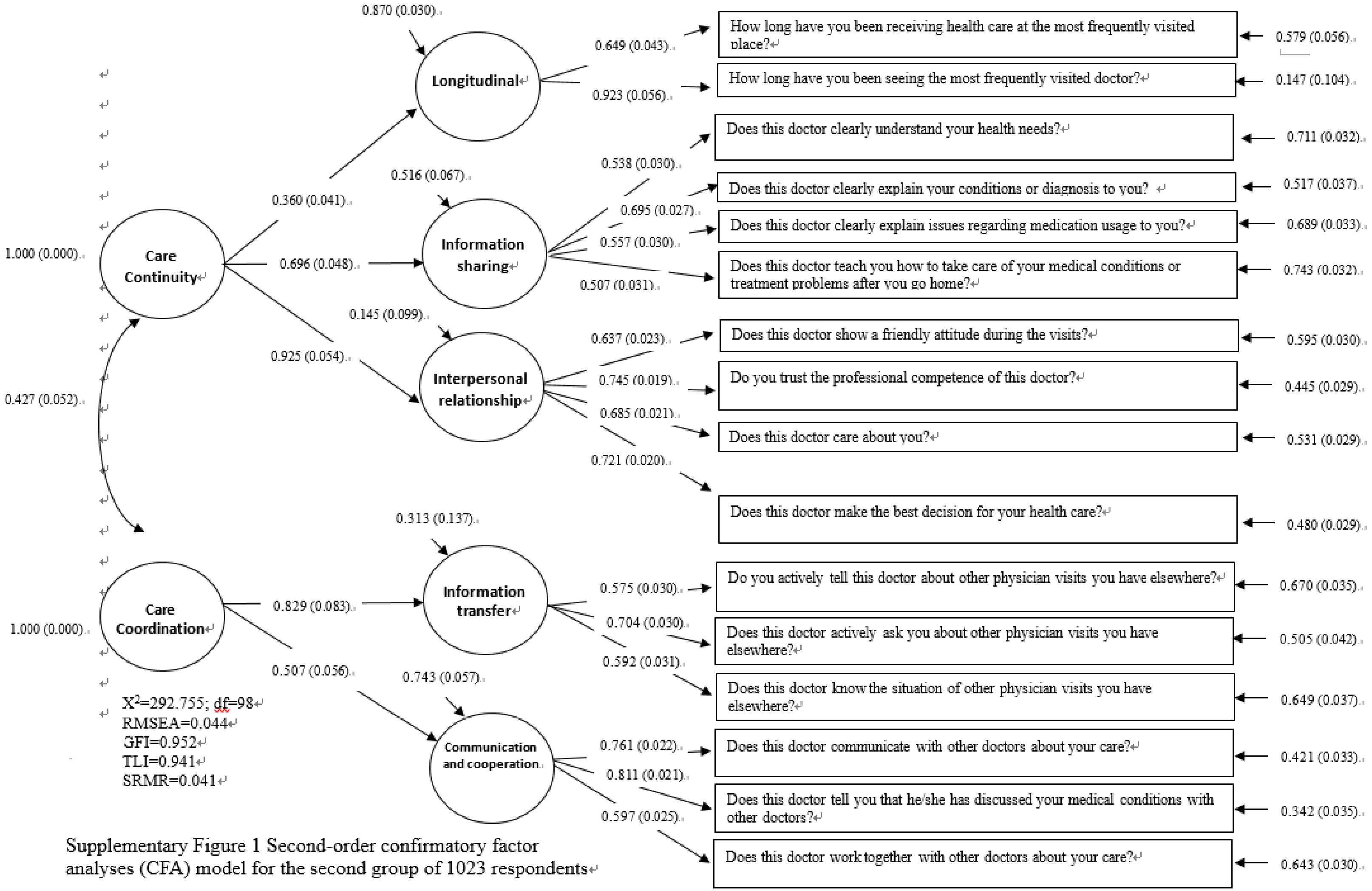

Supplementary Figure 1 Second-order confirmatory factor analyses (CFA) model for the second group of 1023 respondents
